# The fibularis longus muscle revisited: comparative anatomy, developmental perspectives, and clinical relevance

**DOI:** 10.3389/fcell.2025.1678965

**Published:** 2025-10-03

**Authors:** Ingrid C. Landfald, Magdalena Ciechanowska, Łukasz Olewnik

**Affiliations:** ^1^ Department of Clinical Anatomy, Mazovian Academy in Płock, Płock, Poland; ^2^ VARIANTIS Research Laboratory, Department of Clinical Anatomy, Mazovian Academy in Płock, Płock, Poland

**Keywords:** fibularis longus tendon, anatomical variation, classification, fetal anatomy, ultrasound, MRI, surgical relevance, tendoscopy

## Abstract

**Background:**

The fibularis longus tendon (FLT) shows substantial anatomical variability, yet its clinical and developmental implications are incompletely characterised. Classification systems derived from fetal and adult morphology may improve diagnostic interpretation and surgical planning.

**Objective:**

To synthesise comparative, ontogenetic, radiological and surgical perspectives on the FLT into a unified, classification-aware framework.

**Materials and methods:**

Anatomical data from two previously published cohorts—one fetal (n = 94 lower limbs) and one adult (n = 100 lower limbs)—were reviewed to evaluate distal insertion morphotypes. Findings were correlated with imaging literature (MRI, ultrasound) and appraised for diagnostic pitfalls and practical applicability.

**Results:**

Across fetal and adult material, three principal distal insertion types were identified: Type I (single insertion), Type II (bifurcated) and Type III (trifurcated in fetuses or fusion variants in adults). Trifurcated Type III appears confined to prenatal specimens, whereas adult Type III reflects secondary postnatal fusion with neighbouring structures. Radiological correlation highlights recurring misinterpretations involving accessory bands and fusion patterns. Classification-aware interpretation suggests that surgical risk can differ by FLT type, particularly in tendoscopic assessment and tendon transfer planning.

**Conclusion:**

A unified classification aligning fetal and adult variants provides a clinically relevant scaffold for preoperative planning, radiological reporting and anatomical research. Consistent recognition and reporting of FLT subtypes may reduce diagnostic error, inform procedural strategy and enhance anatomical education.

## 1 Introduction

The fibularis longus muscle (FLM) is a key component of the lateral compartment of the leg, originating from the proximal fibula and inserting into the lateral tubercle of the first metatarsal and medial cuneiform. Functionally, it plays a critical role in foot eversion, stabilization of the medial longitudinal arch, and plantar flexion of the ankle (Moore et al., 2017; Thomas et al., 2019). Its unique anatomical course, which includes a strong tendon wrapping beneath the foot from lateral to medial, gives it a mechanical advantage in maintaining foot posture, especially during gait and stance (Bohne et al., 1997; Maynou et al., 2017).

Despite its functional and biomechanical significance, the FLM has been relatively underrepresented in standard anatomical curricula and atlases, often overshadowed by more commonly discussed muscles such as the tibialis posterior or gastrocnemius. Recent imaging and cadaveric studies, however, have highlighted its high morphological variability, especially at the level of its insertion and its accessory structures (Lohrmann et al., 1997; Draghi et al., 2018; Lui and Hau, 2018; Koh et al., 2019; Olewnik, 2019; Olewnik et al., 2022).

The presence of anatomical variants, such as bifurcated or trifurcated insertions and frenular ligaments (anterior - AFL) and posteriori–PFL), has been confirmed by several authors (Patil et al., 2007; Shyamsundar et al., 2012; Olewnik, 2019; Olewnik et al., 2022). These variations may contribute to clinical syndromes such as fibularis tendon instability, tendinopathy, or deformities such as pes cavus, especially in neuromuscular disorders like Charcot-Marie-Tooth disease (Tynan et al., 1992; Berciano et al., 2011).

From an evolutionary and developmental perspective, the FLM has been noted to exhibit phylogenetic plasticity, with reports of anomalous insertions and muscular interconnections dating back to classical works (Picou, 1894a; Picou, 1894b;Wright et al., 1946). Embryologically, the fibularis longus tendon (FLT) forms through lateral mesodermal condensation, and subtle insertional variants may arise during this early developmental stage (Olewnik et al., 2022).

The aim of this study is to provide a comprehensive reassessment of the FLM by integrating data from developmental anatomy, comparative cadaveric research, and clinical imaging. We propose a unified anatomical and morphological classification of FLT insertions, examine their ontogenetic implications, and discuss their relevance to clinical diagnostics and surgical interventions.

## 2 Developmental anatomy

### 2.1 Embryological origin: differentiation from the lateral muscle mass of the lower limb bud

The FLM originates from the lateral mesodermal condensation of the lower limb bud, forming part of the lateral muscle group during embryogenesis. This differentiation is directed by segment-specific gene expression patterns and limb morphogen gradients that define compartmental muscle fate (Moore et al., 2017).

### 2.2 Early tendon formation and anatomical pathway

The FLT emerges early in development, assuming its characteristic course along the lateral leg and posterior to the lateral malleolus, ultimately wrapping beneath the foot to reach the medial cuneiform and first metatarsal base. This unique path is topographically established *in utero* and is essential for its role in foot eversion and longitudinal arch suport (Thomas et al., 2019).

### 2.3 Morphological variability detected in fetal studies

Contrary to the historical assumption of anatomical stability during prenatal development, morphological diversity of the FLT has been confirmed in human fetuses. Olewnik et al. (2022) demonstrated that different insertional patterns, including bifurcated and trifurcated terminations, occur already during fetal life. These observations support the hypothesis that such variation is not solely a postnatal adaptation but may originate *in utero*.

### 2.4 Comparison with the plantaris muscle: developmental consistency vs variability

Unlike the plantaris muscle, which typically shows postnatal variation but maintains prenatal morphological consistency, the FLM demonstrates distinct variability already in fetal specimens (Testut, 1884; LeDouble, 1897; Olewnik et al., 2022). This distinction suggests that the FLM follows a more divergent ontogenetic pathway.

### 2.5 Theoretical embryological mechanisms of variation

Proposed mechanisms for this variability include.

• Segmental differentiation anomalies
• Tendinous migration errors
• Fascial-mesenchymal interaction

### 2.6 Olewnik’s fetal classification

Olewnik et al. (2022) classified the FLT into three main types based on observations from 94 fetal limbs (56 female, 38 male) (Olewnik et al., 2022).

• Type I–Single distal attachment (67 limbs, 71.3%):
  ○ Subtype Ia: insertion to the lateral tubercle of the base of the first metatarsal bone (49 cases).
  ○ *Subtype Ib*: insertion to the head of the first metatarsal bone (18 cases).
• Type II–Bifurcated distal attachment (23 limbs, 24.5%):
  ○ Subtype IIa: main band to the base of the first metatarsal and accessory band to the medial cuneiform (17 cases).
  ○ *Subtype IIb*: complex bifurcation including accessory slips to the fourth dorsal interosseous muscle (4 cases).
  ○ *Subtype IIc*: accessory insertion to the first dorsal interosseous muscle (2 cases).
• Type III–Trifurcated distal attachment (4 limbs, 4.2%):
  ○ Main band to the base of the first metatarsal, with two accessory bands inserting to the medial cuneiform and the first dorsal interosseous.

## 3 Anatomical variability and classification of the FLT

### 3.1 Variations in proximal and distal attachments

The FLM show notable anatomical variability, particularly in the configuration of the distal insertion and the pattern of the proximal muscular origin. In classical descriptions, the FLT inserts via a single tendinous band onto the lateral aspect of the base of the first metatarsal and the medial cuneiform (Moore et al., 2017). Numerous anatomical studies have documented deviations from this pattern. Early reports by Picou (1894b) and Le Double (Picou, 1894b) identified accessory tendinous slips inserting not only into the head or shaft of the first metatarsal, but also into the second metatarsal and into intrinsic plantar muscles such as the dorsal and plantar interossea. More recent investigations have confirmed additional fibrous bands inserting into structures such as the metatarsal–cuneiform joint capsule, the first dorsal interosseous muscle and the plantar aponeurosis; bifurcation or trifurcation of the tendon has been observed in both adult and fetal specimens, suggesting early developmental origins with postnatal persistence (Olewnik, 2019; Olewnik et al., 2022).

Proximally, variations in muscular origin include accessory fascicles or supernumerary heads arising from the lateral surface of the fibula, the anterior or posterior intermuscular septum, or in close relation to fibularis brevis (Macalister, 1875; LeDouble, 1897; Thomas et al., 2019). In some cases these arrangements are associated with partial or complete muscular fusion within the lateral crural compartment, which may complicate purely anatomical interpretation.

In summary, these variants reflect the morphological diversity of the fibularis longus muscle–tendon unit and should be recorded and interpreted within a structured anatomical framework. Clinical implications are summarised in Section 6; see Table 5 for variant-specific mapping and Table 6 for the intraoperative checklist.

### 3.2 Reports of accessory heads, duplicated tendons, and fusion with fibularis brevis

Multiple anatomical reports describe uncommon morphologies of the FLM, including accessory muscular heads, duplicated tendons, and partial or complete fusion with neighbouring muscles, particularly fibularis brevis. Early catalogues by Macalister (1875) documented accessory bellies arising independently or in continuity with fibularis brevis, with origins from the fibula or adjacent intermuscular septa. LeDouble (1897) expanded this spectrum, noting supernumerary FLM heads from variable sites such as anterior or posterior fibular surfaces and the anterior intermuscular septum, and occasional fusion of these slips with fibularis brevis.

Wright and colleagues provided a systematic account of a tendinous variation in which an accessory slip diverged from the main FLT to insert into the first dorsal interosseous muscle, observed in 5 of 125 dissected lower limbs (Wright et al., 1946). Such configurations highlight the potential for complex interconnections across the dorsum and lateral-plantar compartments of the foot.

Contemporary studies using cadaveric dissection and imaging have corroborated these classical observations. Thomas et al. (2019) reported tendon duplication and cross-connections between the FLT and the fibularis brevis tendon, features that can be delineated on high-resolution MRI and confirmed in dissection. A rare case of partial rupture within a duplicated FLT further illustrates the structural variability of distal tendinous arrangements (Koh et al., 2019). Additional work has described fusion planes or connective slips from FLT toward tibialis posterior or adductor hallucis, broadening the catalogue of variant distal relationships (Olewnik, 2019; Edama et al., 2020).

Clinical implications of this anatomical spectrum are summarised in Section 6; see Table 5 for variant-specific risk mapping and Table 6 for intraoperative steps.

### 3.3 Previous classifications of the fibularis longus tendon

Before the structured classification proposed by Olewnik (2019), several authors described anatomical variations of the FLT insertion based on dissection findings. These early typologies were often descriptive, lacked standardization, and varied in anatomical precision. However, they laid the groundwork for subsequent classification efforts.

#### 3.3.1 Picou

In one of the earliest comprehensive anatomical studies, Picou reported various insertion points of the FLT on the plantar surface of the foot. These included (Picou, 1894a).

• The base and head of the first metatarsal bone.
• The medial cuneiform.
• The metatarsocuneiform joint capsule.
• Occasional accessory slips inserting into interosseous muscles.

Although not framed as a classification, Picou’s work established the existence of multiple, functionally distinct insertion zones.

#### 3.3.2 Patil et al.

In a descriptive cadaveric study of 30 lower limbs, Patil et al. (2007) investigated the variability in the distal insertion of the FLT. They observed that while all specimens exhibited insertion to the base of the first metatarsal, a significant proportion also displayed additional slips to the medial cuneiform (86.6%) and accessory attachments near the first tarsometatarsal joint (30%). Further variations included slips to the second, fourth, and fifth metatarsals, as well as to the neck of the first metatarsal. Although no formal classification was proposed, their findings highlighted a wide range of insertional diversity, which holds clinical relevance, particularly in reconstructive procedures and imaging interpretation (Patil et al., 2007).

#### 3.3.3 Shyamsundaret et al.

In their anatomical study based on 40 formalin-fixed lower limbs, Shyamsundar and colleagues proposed a three-type classification system for the distal insertion of the FLT (Shyamsundar et al., 2012).

• **Type I**–Single insertion to the base of the first metatarsal.
• **Type II**–Single insertion to the medial cuneiform.
• **Type III**–Dual insertion to both the base of the first metatarsal and the medial cuneiform.

In addition to these defined types, the authors also observed accessory tendinous slips inserting into.

• The metatarsal–cuneiform joint capsule,
• The interosseous dorsalis muscles, and
• Occasionally, into fibrous tissue of the plantar aponeurosis.

This work significantly expanded upon previous descriptive findings (e.g., Patil et al. (2007)) by establishing a reproducible classification framework. Although they did not assign subtype labels, their documentation of accessory bands and variant insertions emphasizes the need for further morphological refinement.

#### 3.3.4 Summary

These earlier efforts contributed valuable data but shared limitations.

• Lack of internal subdivisions (e.g., subtypes).
• Absence of morphometric criteria.
• No integration with embryological or radiological data.

The classification by Olewnik (2019) was the first to unify anatomical, clinical, and structural aspects in a reproducible system, forming the basis for expanded application in surgical anatomy and developmental studies.

### 3.4 Olewnik’s adult classification

In a comprehensive anatomical study of adult lower limbs, Olewnik (2019) proposed a detailed morphological classification of the FLT, grounded in dissection of 100 specimens. This system introduced three primary types, each with anatomically defined subtypes, and focused on the structure and number of distal insertions (Olewnik, 2019).

#### 3.4.1 Type I–Single distal attachment

**FIGURE 1:**
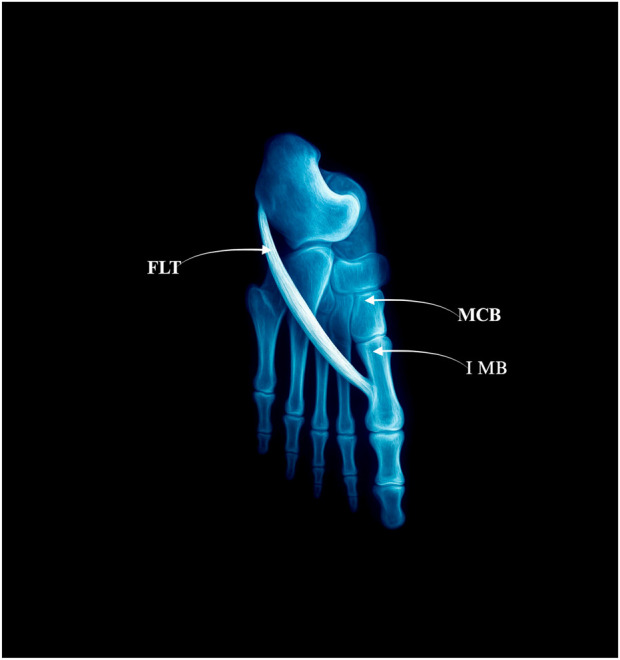
Type I: single distal insertion to the lateral tubercle of the base of the first metatarsal. Abbreviations: FLT fibularis longus tendon; 1 MB the first metatarsal bone. MCB–medial cuneiforme bone.

The FLT inserts solely into the lateral tubercle of the base of the first metatarsal bone–Figure 1.

• Prevalence: 49 limbs (49%)
• No subtypes

#### 3.4.2 Type II–Bifurcated distal attachment

The main tendon inserts into the same lateral tubercle, but additional accessory slips branch off.

**FIGURE 2:**
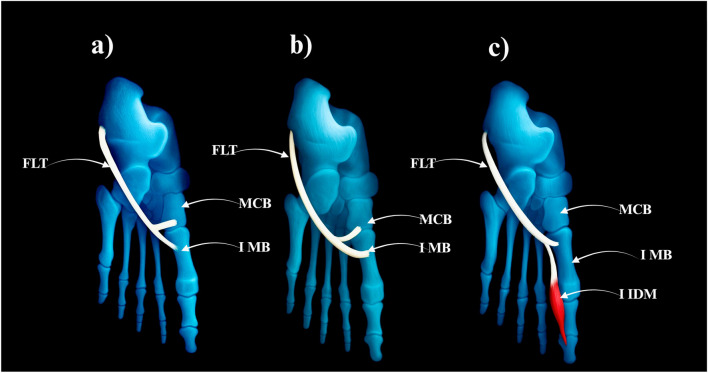
Type II of fibularis longus tendon. **(a)** Type IIa of the fibularis longus tendon. **(b)** Type IIb od the fibularis longus tendon. **(c)** Type IIc od the fibularis longus tendon. FLT–fibularis longus tendon. MCB–medial cuneiforme bone. IMB–first metatarsal bone. I IDM–first dorsal interosseous muscle.

• Prevalence: 40 limbs (40%)
• Subtypes:
  ○ IIa–Accessory slip to the medial cuneiform (28 cases) – Figure 2a.
  ○ **IIb**–Strong accessory slip to both the base of the first metatarsal and the medial cuneiform, involving also the first metatarsal-cuneiform joint (9 cases) = Figure 2b.
  ○ **IIc**–Accessory slip to the first dorsal interosseous muscle (3 cases) – Figure 2c.

#### 3.4.3 Type III–Distal fusion with adjacent tendons

A single FLT that fuses distally with other muscles before inserting.

**FIGURE 3:**
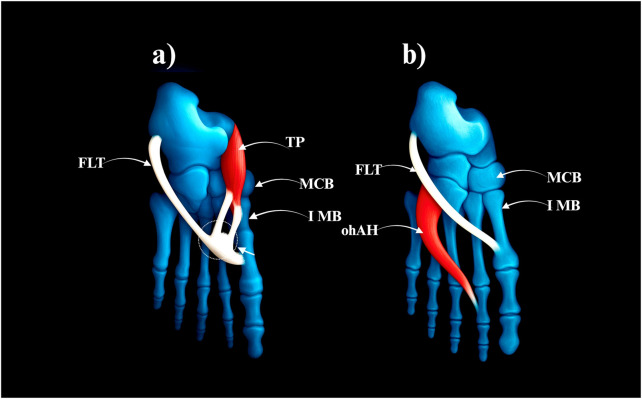
Type III of the fibularis longus tendon. **(a)** Type IIIa fibularis londus tendon. **(b)** Type IIIb fibularis longus tendon. FLT–fibularis longus tendon. TP–tibialis posterior tendon. MCB–medial cuneiforme bone. IMB–first metatarsal bone. ohAH–oblique head of the adductor hallucis.

• Prevalence: 11 limbs (11%)
• Subtypes:
  ○ **IIIa**–Fusion with the posterior tibialis tendon (8 cases) – Figure 3a.
  ○ **IIIb**–Fusion with the adductor hallucis (3 cases) – Figure 3b.

This classification system complements and refines previous descriptive typologies by Patil et al. (2007) and Shyamsundar et al. (2012), introducing a reproducible structure with clearly illustrated insertional patterns. Notably, it is also the first to identify fusion variants **(Type III),** which carry clinical implications in tendon harvesting and imaging misinterpretation.

Compared with the fetal typology by Olewnik et al. (2022), certain adult variants (e.g., **Type IIIb**) appear exclusive to the postnatal period, while bifurcated and single insertions show morphologic continuity across development.

A comparative overview of these classification systems is presented in Table 1.

**TABLE 1:** Comparative overview of FLT classifications.

Author(s)	Year	Classification	Types/Subtypes	Description
Picou	1894	Descriptive	—	Various distal insertions including accessory slips; no formal classification
Patil et al.	2007	Descriptive	A–D (inferred)	Insertions to base of 1st metatarsal, medial cuneiform, and other metatarsals; no named types
Shyamsundar et al.	2012	Formal	Type I–III	Type I: base of 1st MT; Type II: medial cuneiform; Type III: dual insertion
Olewnik et al.	2019	Formal	Type I–III (IIIa–b)	Type I: single; Type IIa–c: bifurcated; Type IIIa–b: trifurcated with fusion variants
Olewnik et al.	2021	Formal (fetal)	Type I–III (Ia–b, IIa–c)	Ia–b: single; IIa–c: bifurcated; III: trifurcated (exclusive to fetuses)

### 3.5 Frenular ligament

The frenular ligament is a specialised fibrous structure related to the FLT, most consistently observed at the level of the cuboid. It has been described in detail in relatively few anatomical studies, notably by Patil et al. (2007) and colleagues, by Olewnik (2019), and by Olewnik and co-authors (Olewnik et al., 2022), and is frequently omitted from classical textbooks (Patil et al., 2007; Olewnik, 2019; Olewnik et al., 2022).

In the most comprehensive adult series, Olewnik proposed two subdivisions based on topography and insertion. The anterior frenular ligament was recorded in 49 of 94 limbs and forms a band that joins the tendon to the fifth metatarsal and the third plantar interosseous muscle, passing anterior to the tendon and potentially guiding its medial rotation beneath the foot. The posterior frenular ligament, present in nine cases, extends from the tendon posteriorly toward the long calcaneocuboid ligament and represents a more vertically oriented fascial connection (Olewnik, 2019).

Subsequent work on fetal material confirmed the presence of both anterior and posterior frenular ligaments, indicating prenatal formation and arguing against a purely postnatal mechanical origin (Olewnik, 2019). This developmental evidence elevates their anatomical importance and supports inclusion of these connections in standard descriptions of the lateral plantar tunnel.

Reported frequencies vary across studies. Patil et al. (2007) first noted frenular structures in adults, identifying the anterior ligament in 25 of 30 specimens and the posterior in 4 of 30, although without detailed insertions. In adults studied by Olewnik (2019), the anterior ligament occurred in 49 of 100 limbs and the posterior in 9 of 100, with defined attachments and relations. In fetal series, the anterior ligament was present in 15 of 94 limbs and the posterior in 6 of 94. The relationship between frenular occurrence and distal FLT morphology is summarised in Table 2, and a cross-study comparison appears in Table 3.

**TABLE 2:** Distribution of frenular ligaments by FLT type (Olewnik, 2019).

FLT Type	Number of Limbs	Anterior Frenular Ligament (AFL)	Posterior Frenular Ligament (PFL)
Type I	49	32	4
Type II	40	13	3
Type III	11	4	2
Total	100	49	9

**TABLE 3:** Comparative presence of frenular ligaments across studies.

Study	Sample Type	Anterior Frenular Ligament (AFL)	Posterior Frenular Ligament (PFL)
Patil et al. (2007)	Adults	25/30 (83.3%)	4/30 (13.3%)
Olewnik (2019)	Adults	49/100 (49%)	9/100 (9%)
Olewnik et al. (2022)	Fetuses	15/94 (16%)	6/94 (6.4%)

Clinical implications and operative handling of frenular restraints are addressed in Section 6; see Table 5 for variant-specific risk mapping and Table 6 for stepwise intraoperative considerations.

A comparative summary of the frequency and distribution of anterior and posterior frenular ligaments reported in these studies is presented in Table 3.

## 3.6 Clinical implications

The anatomical presence and variability of frenular ligaments have practical implications.

• Fibular tendoscopy and lateral foot decompression may be influenced by these bands.
• Entrapment syndromes involving the FLT may involve fibrous restraint from these structures.
• During tendon graft harvesting, frenular ligaments may complicate dissection or require careful release.

Despite limited clinical literature, the anatomical evidence justifies routine consideration of these structures during both imaging interpretation and operative planning.

## 4 Evolutionary, developmental and comparative anatomy of the fibularis longus muscle

### 4.1 Evolutionary background

The FLM originates from the posterior muscle mass of the lower limb bud, a mesodermal structure that remains largely undifferentiated in early tetrapods such as amphibians. In these primitive forms, lateral leg muscles are not organized into defined compartments, and their role in foot eversion is minimal.

With the transition to terrestrial locomotion, reptiles and other early amniotes developed more specialized musculature along the lateral compartment of the leg. These evolutionary innovations included the emergence of tendon-forming lateral muscles that contributed to mediolateral stability during sprawling gait (Abdala and Diogo, 2010).

In birds, lateral leg muscles became further specialized to accommodate perching and grasping mechanics, often forming fibrous insertions with variable homology to mammalian fibular structures (Diogo et al., 2008). Although some remnants of the fibularis group persist in avian species, the human FLM trajectory with a deep plantar course is not observed.

In mammals, particularly in primates, the FLM gained new biomechanical relevance through the evolution of plantigrade locomotion and the development of a structurally significant medial longitudinal arch. This required a strong dynamic stabilizer of the first ray and the transverse arch, functions now performed by the FLM and its inferoplantar tendon course (Diogo and Wood, 2012).

The complete rotation of the lower limb during hominin evolution further emphasized the FLM’s role in supporting upright posture and complex foot mechanics. The increased morphological diversity of its insertion patterns, including bifurcation and fusion with intrinsic plantar structures, may reflect adaptive responses to bipedal loading (Diogo and Wood, 2012).

Understanding this evolutionary trajectory from undifferentiated lateral musculature in early tetrapods to a specialized arch-stabilizing structure in humans offers critical context for interpreting anatomical variability and clinical relevance of the FLT.

### 4.2 Variational trends in phylogeny and ontogeny

Muscular and tendinous variability of the FLM can be interpreted as a product of both evolutionary and developmental processes. Across vertebrate lineages, the transition from a generalized lateral muscle mass to a distinct fibularis group has occurred in parallel with diversification in limb posture, gait, and foot architecture (Abdala and Diogo, 2010; Diogo and Molnar, 2014).

In lower vertebrates such as amphibians and early reptiles, the absence of compartmentalized lateral musculature correlates with limited specialization in tendon insertion. With the emergence of more derived tetrapods, including mammals and especially primates, the FLM begins to show increasing morphological complexity, including differentiated tendon trajectories and variable distal insertions (Diogo and Abdala, 2010).

Ontogenetically, the development of the FLT appears to follow a conservative trajectory during early gestation. However, anatomical studies indicate that fetal variants of distal insertion such as bifurcated or trifurcated tendons may appear earlier than their adult counterparts. These findings suggest that some variants are established during embryogenesis, while others may regress or remodel during late gestation or postnatally (Olewnik et al., 2022).

The fetal classification proposed by Olewnik et al. (2022), based on dissection of 94 fetal lower limbs (56 female and 38 male), identified three major types and multiple subtypes based on the morphology and insertion patterns of the FLT.

**FIGURE 4:**
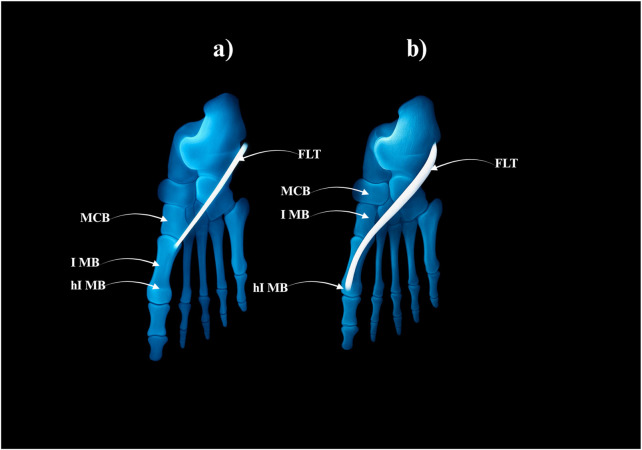
Type I of the fibularis longus tendon. **(a)** Type Ia of the fibularis longus tendon. **(b)** Type Ib of the fibularis longus tendon. MCB–medial cuneiforme bnone. I MB–first metatarsal bone hI MB–head of the first metatarsal bone. FLT–fibularis longus tendon.

Type I–Single Distal Attachment (71.3% of cases; 67/94).

• Subtype Ia: Tendon inserts into the lateral tubercle of the base of the first metatarsal bone (49 cases) – Fig, 4a.
• Subtype Ib: Tendon inserts into the head of the first metatarsal bone (18 cases) – Figure 4b.

Type II–Bifurcated Distal Attachment (24.5% of cases; 23/94).

**FIGURE 5:**
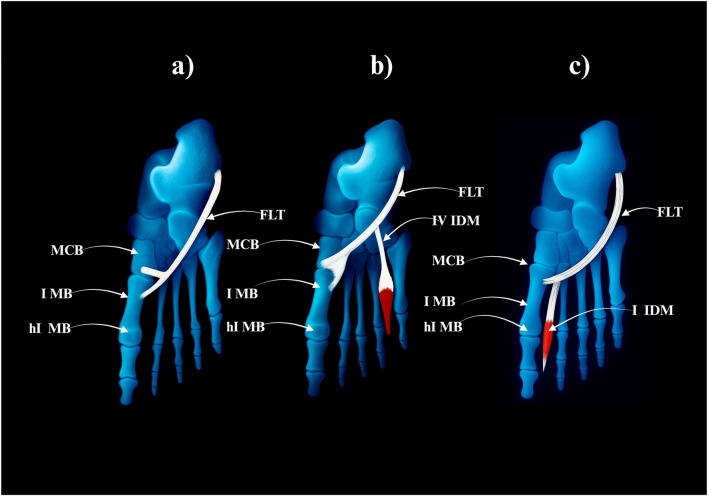
Type II of the fibularis longus tendon. **(a)** Type IIa of the fibularis longus tendon. **(b)** Type IIb of the fibularis longus tendon. **(c)** Type IIc of the fibularis longus tendon. FLT–fibulars longus tendon. MCB–medial cuneiforme bone. I MB–first metatarsal bone. hI MB–head of the first metatarssal bone. IV IDM - fourth interosseus dorsalis muscle. I IDM–first interosseus dorsalis muscle.

• Subtype IIa: Main tendon inserts into the lateral tubercle of the base of the first metatarsal bone; accessory band inserts into the medial cuneiform (17 cases) – Figure 5a.
• Subtype IIb: Strong accessory band inserts into the base of the first metatarsal and medial cuneiform bones, and additional slips attach to the fourth interosseus dorsalis muscle (4 cases) – Figure 5b.
• Subtype IIc: Accessory band inserts into the first interosseus dorsalis muscle (2 cases) – Figure 5c.

Type III–Trifurcated Distal Attachment (4.2% of cases; 4/94).

**FIGURE 6:**
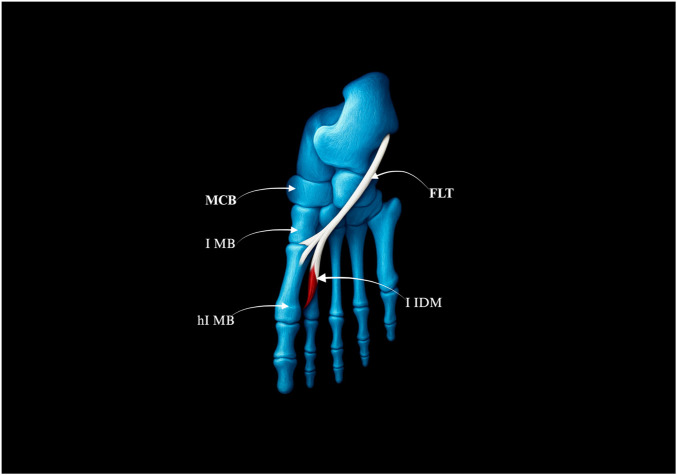
Type III of the fibularis longus tendon. FLT–fibularis longus tendon. I IDM - first interosseus dorsalis muscle. MCB–medial cuneiforme bone. I MB–first metatarsal bone. hI MB–head of the first metatarsal bone.

• The main tendon inserts into the base of the first metatarsal bone; the first accessory band into the medial cuneiform bone, and the second into the first interosseus dorsalis muscle–Figure 6.

These morphotypes show partial overlap with the adult classification proposed by Olewnik (2019), though the trifurcated Type III appears exclusive to the prenatal period. Such developmental restriction may reflect transient morphogenetic events, such as temporary retention or remodeling of accessory bands. Taken together, this system provides a morphologically exhaustive classification that lays the foundation for understanding FLT variability across the human lifespan.

### 4.3 Comparative anatomy across taxa

Comparative studies across vertebrate taxa demonstrate substantial differences in the presence, form, and insertion of the FLM. These differences are closely aligned with species-specific adaptations in limb posture, gait dynamics, and foot morphology (Abdala and Diogo, 2010; Diogo and Abdala, 2010).

In amphibians, the musculature of the lateral leg remains relatively undifferentiated. No direct analog of the FLM is observed, and the primitive muscular structures in this region show limited functional specialization. This reflects the relatively simple demands of aquatic or semi-terrestrial locomotion (Diogo and Abdala, 2010).

Among reptiles, partial differentiation of lateral leg muscles begins to appear. While some elements may be homologous to the FLM, these muscles often insert more proximally and are not involved in arch support. Their function is primarily stabilizing during sprawling gait, with minimal contribution to plantar dynamics (Diogo, 2007).

In birds, elements of the fibularis group are either reduced or highly modified. In many species, the FLM is vestigial or incorporated into complex tendon systems adapted for perching and grasping. The typical inferoplantar trajectory of the human FLT is not replicated in avian species (Diogo and Abdala, 2010).

Among mammals, greater variability in the FLM is noted. In quadrupeds such as canids and ungulates, the FLM is generally present but displays differences in muscle mass, tendon length, and insertion targets. Its role in these species is limited to lateral stabilization of the foot and ankle rather than maintenance of a plantar arch (Yammine and Eric, 2015).

In non-human primates, a shift in FLM morphology becomes apparent, paralleling the emergence of plantigrade locomotion and enhanced grasping abilities. The FLT becomes longer and often inserts more medially, a trait especially pronounced in apes (Diogo and Wood, 2012). However, full inferoplantar translocation of the tendon is rare outside the human lineage.

In Homo sapiens, the FLM reaches its most derived form. The tendon passes under the cuboid in a groove (sometimes forming a peroneal tunnel), then courses medially to insert on the base of the first metatarsal and medial cuneiform. This unique trajectory forms a dynamic sling that supports the transverse and medial longitudinal arches critical for upright bipedal gait (Diogo and Wood, 2012).

Thus, the FLM exemplifies the evolutionary transition from a general-purpose lateral leg muscle to a specialized arch stabilizer in bipedal humans. Its interspecies variability highlights how morphology reflects biomechanical function, offering a compelling model for evolutionary adaptation in musculoskeletal design.

#### 4.3.1 Mammalian trends and functional correlates

Among mammals, the FLM exhibits notable variability that reflects locomotor specialization. In arboreal primates, the FLM tends to be more robust and well-developed, supporting grasping and control of the first ray during climbing and suspension (Diogo and Wood, 2012). In contrast, in cursorial quadrupeds such as canids or ungulates, the FLM is often reduced, fused with adjacent muscles, or exhibits shortened tendons. These species rely more on sagittal plane propulsion than lateral foot control, which reduces the functional necessity of an independent FLM (Diogo and Abdala, 2010; Yammine and Eric, 2015).

In humans, the FLM reaches its most elaborate form long tendon, inferoplantar trajectory, and dual insertion reflecting the dual requirement for arch stabilization and first ray control during bipedal locomotion. This functional differentiation aligns with the evolutionary shift from generalized grasping to terrestrial weight-bearing.

The trend supports the hypothesis that FLM morphology is a functional indicator of limb usage.

• Grasping primates → strong FLM for medial control
• Running quadrupeds → regressed or fused FLM
• Humans → specialized FLM for bipedal stability

**FIGURE 7:**
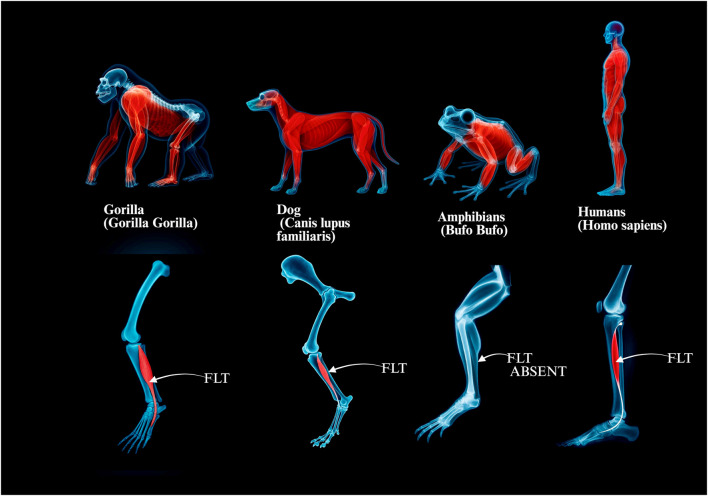
Comparative anatomy of the fibularis longus across selected vertebrate groups. Panels illustrate the presence, relative development and course of the fibularis longus muscle–tendon unit (FLT) from non-human primates and quadrupeds to amphibians and humans: a, *Gorilla gorilla* (robust fibularis longus; relatively short plantar course). b, *Canis lupus* familiaris (reduced FLM/FLT; limited plantar trajectory). c, Anuran amphibian (e.g., *Bufo bufo*), FLT absent. d, *Homo sapiens* (long inferoplantar tendon course supporting the medial and transverse arches). Abbreviation: FLT fibularis longus tendon.

To visually summarise the evolutionary trends in FLT morphology and function, a schematic overview of key vertebrate taxa is provided (Figure 7). It demonstrates both anatomical diversification and functional specialisation of the FLT, culminating in the adapted configuration found in modern humans; see Table 4.

**TABLE 4:** Comparative presence, morphology, and function of the fibularis longus muscle in selected mammals.

Species	FL Presence	FL Morphology	Dominant Function	Locomotor Strategy	References
Human (*Homo sapiens*)	Present	Fully developed, long tendon	Arch support, eversion	Bipedal	Diogo and Wood (2012)
Chimpanzee (*Pan troglodytes*)	Present	Robust, grasp-adapted	Grasping, first ray control	Arboreal/terrestrial	Diogo and Wood (2012)
Gorilla (*Gorilla gorilla*)	Present	Robust, similar to human	Grasping, climbing	Arboreal/terrestrial	Diogo and Wood (2012)
Macaque (*Macaca mulatta*)	Present	Well-defined, arboreal type	Grasping, eversion	Arboreal/terrestrial	Diogo et al. (2008)
Orangutan (*Pongo pygmaeus*)	Present	Robust, long tendon	Suspensory grasping	Arboreal	Diogo and Wood (2012)
New World monkey (e.g., Cebus)	Present	Variable, often elongated	Grasping/balancing	Arboreal	Diogo et al. (2008)
Dog (*Canis lupus* familiaris)	Reduced	Shortened, rudimentary	Propulsion	Cursorial	Yammine and Eric, 2015
Cat (*Felis catus*)	Reduced	Shortened, rudimentary	Agility, balance	Digitigrade	Yammine and Eric, 2015
Horse (Equus ferus caballus)	Fused/Minimal	Fused with lateral muscles	Sagittal propulsion	Cursorial	Borthakur et al., 2023
Cow (*Bos taurus*)	Fused/Minimal	Fused with lateral muscles	Weight support, propulsion	Cursorial	Borthakur et al., 2023

### 4.4 Functional interpretation

The FLM acts as a dynamic stabiliser of the foot, contributing to eversion at the subtalar joint and to support of the medial and transverse arches. Its distinctive course beneath the foot from the lateral compartment to the medial cuneiform and first metatarsal allows a plantarflexory and pronatory influence on the first ray during stance and propulsion (Bohne et al., 1997). This sling-like mechanism helps lock the midfoot at toe-off and supports efficient forward progression in bipedal gait; by modulating first metatarsal position it may also counter excessive medial drift (Thomas et al., 2019).

Morphological variation in the FLM, particularly in distal insertion patterns, can modulate these mechanics. Bifurcated insertions to the medial cuneiform or intrinsic muscles (for example, the first dorsal interosseous) may alter force vectors or redistribute load across plantar structures, while connective relationships or fusions with neighbouring muscles such as tibialis posterior or adductor hallucis may adjust tendon trajectory and tension (Olewnik, 2019; Edama et al., 2020).

Anatomical diversity in FLM–FLT has been associated with differing foot kinematics in selected conditions. In pes cavus related to neuromuscular disease, hypertrophy or altered insertions have been discussed as potential contributors to first-ray plantarflexion and elevated arches; conversely, attenuation or rupture of the FLT has been linked to impaired forefoot control in some reports (Berciano et al., 2011; Koh et al., 2019). Frenular ligaments at the cuboid level may also influence tendon guidance and resistance to shear, with reported variability across studies and possible relationships to distal insertion type (Patil et al., 2007; Olewnik, 2019).

In summary, the FLM–FLT unit is biomechanically specialised and exhibits meaningful anatomical variability that can shape foot motion and load sharing. Procedure-oriented guidance is presented in Section 6; variant-to-pitfall mapping appears in Table 5 and a stepwise intraoperative checklist in Table 6.

**TABLE 5:** FLT variant → imaging pitfall → surgical implication → recommended action.

FLT variant (classification-friendly label)	Typical imaging pitfall	Surgical implication (tendoscopy/open)	Recommended action
Classic single distal slip to medial cuneiform + base of 1st MT	Magic-angle at cuboid tunnel; physiologic flattening in the peroneal groove misread as tendinopathy	Over-debridement of normal tendon/sheath	Use oblique-sagittal MRI and dynamic US to confirm normal contour; avoid aggressive debridement; correlate with symptoms (Wang et al., 2005; Davda et al., 2017)
Bifid/trifid distal FLT (multiple slips)	Interpreted as split tear or synovial septum	Incomplete decompression if only one slip is addressed; residual snapping	Preoperative mapping of all slips; probe and tag slips before release; complete decompression under direct vision (Mota and Rosenberg, 1998; Maffulli et al., 2018; Olewnik, 2019)
Accessory slip to 2nd MT/variable cuneiform insertion	Linear low-signal band misread as scar tissue	Unintentional transection alters plantar vector; suboptimal graft length	Preserve accessory slip when feasible; if harvesting FL, consider partial preservation or alternative graft; document variant in the operative note (Olewnik, 2019)
Fusion/connection with tibialis posterior (TP)	Confluent tendon signal with TP; unclear separation on short-axis MRI	Risk of TP sheath violation; potential TP dysfunction if misidentified	Plan medial exploration if needed; protect the TP sheath; avoid blind division; reconsider FL harvest if fusion is extensive (Olewnik, 2019)
Fusion/connection with adductor hallucis (AH)	Plantar low-signal band mistaken for plantar plate	Release may change hallux mechanics; medial forefoot pain	If release is required, perform incremental division with functional testing; prefer partial preservation (Olewnik, 2019)
Prominent frenular restraints (AFL/PFL)	Linear bands mimicking scar; anisotropy on US	Tethering leading to persistent snapping after incomplete release; iatrogenic injury if unrecognised	Identify AFL/PFL; selective release under direct vision; avoid blind shaver work near restraint (Olewnik, 2019; Lui and Hau, 2018)
Low-lying muscle belly/hypertrophic peroneal tubercle	Crowding causing pseudo-tenosynovitis on MRI/US	Portal crowding; instrument conflict; incomplete synovectomy	Adapt portal placement; gentle sheath dilation; consider limited retinacular release (Davda et al., 2017; Lui and Hau, 2018)
Split FLT within common sheath (with fibularis brevis nearby)	Mislabelled as split-brevis tear	Wrong tendon treated; persistent symptoms	Dynamic US to identify independent excursion; intraoperative traction test to confirm identity before debridement (Maffulli et al., 2018; Davda et al., 2017)
High-grade synovitis with accessory septa	Septum read as partial tear	Blind shaver injury; incomplete symptom relief	Blunt probe first; resect septum under direct view; reassess excursion (Lui and Hau, 2018)

**TABLE 6:** Imaging → intraoperative checklist for FLT tendoscopy/open surgery.

Step	Preoperative MRI/US cue	Intraoperative verification	Pitfall to avoid	Practical note
1. Identify variant	Multiple distal slips? Fusion signs with TP/AH? Frenular bands?	Probe along course; tag each slip with suture	Treating one slip only; cutting fused TP/AH	Cross-check with classification scheme cited in text (Olewnik, 2019)
2. Portal planning	Crowding at peroneal groove/cuboid tunnel	Trial instrument passage before shaver	Forcing shaver leading to sheath/retinaculum injury	Use a more distal working portal for crowding; keep the scope in a safe triangle (Lui and Hau, 2018)
3. Confirm identity	Dynamic US: differential excursion FL vs. fibularis brevis	Traction and visual confirmation	Debriding the wrong tendon	Gentle traction test; trace plantar course of FL.
4. Address restraints	MRI/US hints of AFL/PFL	Visualise the band before division	Blind release causing iatrogenic cut	Cut under direct vision; reassess glide (Olewnik, 2019)
5. Decide on harvest	Variant affects vector/length?	Trial mobilisation; measure obtainable length	Insufficient graft length; arch dysfunction	Consider partial preservation or alternate graft if fusion is extensive
6. Final check	Post-release free glide on scope/US	Passive ROM with visualised excursion	Residual snapping from missed slip	Re-inspect proximally and distally before closure

### 4.5 Clinical and surgical implications

This subsection summarises anatomical considerations only; detailed procedure-oriented guidance is consolidated in Section 6. Variability in distal insertions, accessory slips and occasional fusion with neighbouring muscles (notably fibularis brevis, tibialis posterior and adductor hallucis) creates a spectrum of tendon courses and fascial relationships along the lateral and plantar aspects of the foot. These patterns influence how the FLT may appear on imaging and where fascial reinforcements or connective slips are expected to occur (Patil et al., 2007; Olewnik, 2019; Thomas et al., 2019; Olewnik et al., 2022).

At the level of the cuboid and lateral column, the tendon may be accompanied by fascial bands described as frenular ligaments, with anterior and posterior variants reported in adult and fetal series, respectively (Patil et al., 2007; Olewnik, 2019; Olewnik et al., 2022). Across historical and contemporary material, reports also include duplicated tendons, cross-connections and variant slips to intrinsic foot muscles, each of which can alter expected topography without implying a uniform functional consequence (Macalister, 1875; LeDouble, 1897; Wright et al., 1946; Olewnik, 2019; Thomas et al., 2019).

Comparative and developmental observations provide context for this spectrum but do not themselves prescribe management pathways. Procedure-oriented guidance is presented in Section 6; variant-to-pitfall-to-action mapping is summarised in Table 5 and the stepwise intraoperative checklist in Table 6.

## 5 Imaging considerations and radiological correlates

### 5.1 MRI characteristics of FLT variants

Magnetic resonance imaging provides high soft-tissue contrast for assessing fibularis longus tendon morphology, including insertional architecture, signal heterogeneity and relationships to the peroneal retinacula, os peroneum and adjacent musculature (Wang et al., 2005; Taljanovic et al., 2015). Bifurcated or trifurcated insertions, partial continuity with tibialis posterior and accessory slips toward intrinsic foot muscles can be visualised as multiple low-signal tendinous bands deviating from the canonical inferoplantar course and attaching to atypical landmarks (Wang et al., 2005; Draghi et al., 2018). MRI also depicts the peroneal tubercle and the retromalleolar groove, landmarks relevant to tendon trajectory and potential entrapment zones; shallow or convex retromalleolar morphology has been associated with subluxation patterns and split tears (Neustadter et al., 2004; Taljanovic et al., 2015). Indirect indicators of variant anatomy include altered muscle-belly volume, oedema-like signal adjacent to accessory bands and tenosynovial thickening, potentially reflecting altered traction vectors (Saupe et al., 2007). Fusion variants involving tibialis posterior or adductor hallucis may appear as continuous low-signal fascicles crossing compartments or demonstrating dual insertional footprints (Bianchi and Martinoli, 2007). The magic-angle effect, particularly along the plantar curve beneath the cuboid, can falsely elevate intratendinous signal and should be considered to avoid overcalling tendinopathy (Saupe et al., 2007). Correlation with dissection-based classification improves interpretive accuracy: Type I usually presents as a single homogeneous low-signal band to the first metatarsal/medial cuneiform complex, whereas Types II and III show multiple slips or fusion zones better appreciated on axial and oblique-sagittal reconstructions (Wang et al., 2005; Olewnik, 2019). Imaging findings relevant to operative decision-making are discussed in Section 6; see Table 5, 6.

### 5.2 Ultrasonographic features and dynamic assessment

Ultrasonography offers real-time evaluation of FLT behaviour during motion and complements MRI for dynamic phenomena such as subluxation, snapping and intrasheath instability (Draghi et al., 2018). On ultrasound the FLT appears as a fibrillar hyperechoic structure deep to fibularis brevis within the superior peroneal retinaculum, with differential axial positioning aiding tendon identification (Taljanovic et al., 2015). Dynamic manoeuvres, including resisted eversion or dorsiflexion with circumduction, can elicit anterior subluxation or intrasheath migration, particularly with shallow retromalleolar grooves or retinacular deficiency; these events may be missed on neutral-position MRI (Mota and Rosenberg, 1998; Neustadter et al., 2004). Accessory slips, bifurcated or trifurcated insertions and fascial bands can be depicted with high-frequency probes, though interpretation requires awareness of anisotropy and careful three-plane scanning; compound imaging and power Doppler can improve conspicuity in selected scenarios (Taljanovic et al., 2015; Davda et al., 2017). Integration of ultrasound with MRI is recommended when fusion variants or slips to intrinsic muscles complicate the sonographic appearance (Davda et al., 2017; Olewnik, 2019). Imaging findings relevant to operative decision-making are discussed in Section 6; see Table 5, 6.

### 5.3 Common diagnostic pitfalls

Variant distal insertions, accessory slips and fusions can mimic pathology or remain unrecognised on routine protocols. Frequent misinterpretations include labelling Type II configurations as fibularis brevis pathology or as longitudinal partial tears; careful tracking of tendon course on orthogonal planes mitigates this risk (Bianchi and Martinoli, 2007; Martinoli et al., 2012; Davda et al., 2017). Ultrasound anisotropy may produce inconsistent echogenicity in oblique fibres and branching slips; omission of longitudinal and coronal sweeps increases the chance of false positives for degeneration or instability (Bordalo et al., 2023). Complex trifurcations or slips into interosseous muscles may be missed without thin-slice, fat-suppressed sequences or targeted dynamic ultrasound (Wang et al., 2005; Olewnik et al., 2022). Adult Type III fusion variants can simulate retinacular or midfoot ligamentous structures unless classification-aware interpretation is applied (Olewnik, 2019). A structured imaging checklist that emphasises tendon continuity tracking, differentiation from fibularis brevis, search for accessory bands and reference to anatomical subtypes improves consistency across readers. Imaging findings relevant to operative decision-making are discussed in Section 6; see Table 5, 6.

### 5.4 Radiological correlation with anatomical classifications

Alignment of imaging descriptors with dissection-based classifications enhances diagnostic precision and creates a shared lexicon with surgeons. The adult classification proposed by Olewnik distinguishes single, bifurcated and fusion patterns that are visible on MRI when multiplanar reconstructions are used; Type I typically shows a single low-signal band, Type II demonstrates additional slips to structures such as the medial cuneiform or intrinsic muscles, and Type III exhibits tendinous continuity across compartments suggestive of Fusion (Wang et al., 2005; Taljanovic et al., 2015; Olewnik, 2019). On ultrasound, operator-dependent but high-resolution, three-plane scanning allows recognition of bifurcated insertions and fascial slips and appreciates dynamic effects produced by accessory components (Davda et al., 2017). Emerging work has begun to address the imaging visibility of fetal variants described by Olewnik and colleagues, although paediatric and prenatal MRI remain technically constrained; *postmortem* and advanced fetal imaging provide proof-of-principle in selected patterns (Olewnik et al., 2022). Integrating classification terminology into radiology reports supports targeted communication and reduces the likelihood of normal variants being interpreted as disease. Imaging findings relevant to operative decision-making are discussed in Section 6; see Table 5, 6.

## 6 Clinical and surgical implications

### 6.1 Relevance in tendoscopy and foot surgery

Anatomical variability of the FLT directly influences both tendoscopy and open foot surgery. To minimise incomplete exploration, misidentification and iatrogenic injury, surgeons should apply a classification-aware strategy; a concise variant → imaging pitfall → surgical implication → recommended action map is provided in Table 5.

Tendoscopy of the fibular (peroneal) tendons is increasingly used for lateral ankle pain, snapping and tenosynovitis (Lui and Hau, 2018). Bifid or trifid insertions and accessory slips can mimic partial tears or synovial septa on arthroscopy and imaging (Mota and Rosenberg, 1998; Maffulli et al., 2018; Olewnik, 2019). Practical responses, including preoperative mapping of all slips, intraoperative tagging and complete decompression, are summarised in Table 5.

Frenular restraints are equally relevant. The accessory frenular ligament may tether the FLT near the fifth metatarsal or cuboid and has been reported in roughly half of adult specimens (Olewnik, 2019). Selective, visualised release and post-release glide assessment are listed in the intraoperative checklist in Table 6, with additional imaging correlates in Section 5.

In open surgery, including lateral retinacular procedures, subtalar stabilisation and tendon transfers, fusion patterns or slips towards neighbouring structures such as tibialis posterior or adductor hallucis alter dissection planes and obtainable graft length. Planning considerations and intraoperative safeguards for these variants are consolidated in Table 5 (Olewnik, 2019; Olewnik et al., 2022).

Preoperative, classification-aware imaging improves planning. MRI and high-resolution ultrasound can anticipate multiple slips, fusions and restraining bands when interpreted with explicit awareness of anatomical diversity (Wang et al., 2005; Davda et al., 2017). Key intraoperative verification steps, including variant identification, portal planning, tendon identity confirmation and post-release glide assessment, are listed in Table 6.

Take-home: use Table 5 for rapid, variant-specific risk mapping and Table 6 as a procedural checklist, and combine both with classification-aware MRI and ultrasound to reduce avoidable errors (Davda et al., 2017; Lui and Hau, 2018; Olewnik, 2019).

## 7 Proposed unified classification framework

Anatomical variation of the FLT has been extensively described in both fetal and adult populations, yet a truly integrative classification system bridging developmental and postnatal morphotypes remains lacking. Existing classifications, such as those by Olewnik (2019), Olewnik et al. (2022), have independently categorized adult and fetal insertions, respectively, but have not been formally synthesized into a unified, clinically applicable model. This section proposes a harmonized classification framework that integrates embryological insights with adult morphology and offers a consistent basis for clinical and radiological interpretation.

### 7.1 Integration of developmental and adult variants

The fetal classification proposed by Olewnik et al. (2022), based on 94 lower limbs, revealed three main types: single insertion (Type I), bifurcated insertion (Type II), and trifurcated insertion (Type III). These were further subdivided based on target structures such as the medial cuneiform and dorsal interosseous muscles. Notably, certain variants particularly the trifurcated Type III were observed exclusively in fetal specimens, suggesting possible regression or remodeling during late gestation or early postnatal life.

The adult classification by the same group (Olewnik, 2019), based on 100 dissected limbs, included a similar Type I (single insertion), Type II (bifurcated), and an additional Type III representing fusion variants with adjacent tendons (e.g., tibialis posterior, adductor hallucis). This divergence underscores a developmental continuum wherein certain fetal insertions may fuse, remodel, or regress, resulting in the adult anatomical spectrum.

By aligning corresponding types across these classifications, a developmental trajectory can be inferred.

• Fetal Type I corresponds directly to Adult Type I.
• Fetal Type II evolves into Adult Type IIa or IIb depending on the maturity and persistence of accessory slips.
• Fetal Type III, involving trifurcation, appears absent in adults, likely due to postnatal tendon refinement or muscular integration.
• Adult Type III (fusion variants) likely develops secondarily, independent of fetal trifurcation.

This developmental–adult alignment reinforces the notion that fetal Type III should not be interpreted as a precursor to adult Type III fusion variants, but rather as a transient morphogenetic pattern without a persistent postnatal equivalent.

### 7.2 Proposed classification key and subtypes

The unified classification includes three major types with corresponding subtypes reflecting both prenatal and postnatal morphology.

#### 7.2.1 Type I–Single insertion

• Ia: Insertion to the base of the first metatarsal (fetal/adult)
• Ib: Insertion to the head of the first metatarsal (fetal)

#### 7.2.2 Type II–Bifurcated insertion

• IIa: Additional slip to the medial cuneiform (fetal/adult)
• IIb: Additional slip to plantar or dorsal interosseous muscles (fetal/adult)
• IIc: Complex bifurcation involving joint capsule (adult only)

#### 7.2.3 Type III–Fusion variant or complex insertion

• IIIa: Fusion with tibialis posterior (adult)
• IIIb: Fusion with adductor hallucis (adult)
• IIId: Persistent trifurcation (fetal only; no adult analog)

This system allows mapping of individual cases from fetal origin through adult morphology and offers an interpretive bridge between ontogeny and clinical presentation. (See Table 7 for a comparative overview.).

**TABLE 7:** Comparative overview of fetal and adult classification systems for the fibularis longus tendon (FLT).

Unified Type	Fetal Classification (Olewnik et al., 2022)	Adult Classification (Olewnik, 2019)	Developmental Interpretation
Type I	- Ia: insertion to lateral tubercle of 1st metatarsal - Ib: insertion to head of 1st metatarsal	- Single insertion to base of 1st metatarsal	Direct fetal-to-adult correspondence
Type II	- IIa: accessory band to medial cuneiform - IIb: bands to medial cuneiform +4th interosseus dorsalis - IIc: band to 1st interosseus dorsalis	- IIa: additional slip to medial cuneiform - IIb: broader bifurcation, sometimes muscular	Partial transformation; selective retention of accessory bands
Type III	- III: trifurcation to 1st metatarsal, medial cuneiform, 1st interosseus dorsalis	- IIIa: fusion with tibialis posterior - IIIb: fusion with adductor hallucis	No continuity; likely postnatal secondary development

### 7.3 Clinical applications of the unified system

The unified classification system combining fetal and adult variants of the FLT serves as a robust framework for guiding clinical decision-making across diagnostic and surgical domains. Its strength lies in the predictive mapping of morphotypes, allowing clinicians to anticipate tendon complexity and individual anatomical nuances prior to intervention.

From a radiological perspective, the system enhances diagnostic accuracy by encouraging a structured search pattern tailored to known subtype features. Awareness of variant-specific insertion sites and fusion patterns reduces misinterpretation of accessory slips, soft-tissue masses, or anomalous signal intensities. As detailed in Section 5.3, common diagnostic pitfalls can be significantly mitigated by referencing anatomical subtype data during imaging analysis (Bianchi et al., 2007).

In surgical settings, the classification offers a template for procedural planning. Recognition of Type II and III variants enables better control of tendon harvest boundaries, guides portal placement in tendoscopy, and prevents inadvertent damage to adjacent neurovascular or muscular structures (Olewnik, 2019).

Furthermore, the classification aids in surgical reporting, inter-specialist communication, and education. Structured terminology improves clarity in operative notes, facilitates longitudinal documentation of rare morphotypes, and supports standardized cadaveric or imaging studies across institutions (Cullen et al., 2017; Olewnik, 2019).

Importantly, the unified system provides a developmental context for adult anomalies, fostering better understanding of how fetal variants contribute or fail to persist into postnatal morphology. This insight is particularly valuable when assessing pathology in adolescents or planning pediatric reconstructions.

The integration of anatomical and ontogenetic classifications, therefore, bridges the gap between embryology, radiology, and surgical anatomy, supporting a more precise, individualized approach to musculoskeletal care.

### 7.4 Validation prospects in imaging and surgery

Validation of this classification framework requires integration into prospective imaging studies and intraoperative registries. High-resolution MRI, combined with intraoperative video documentation or cadaveric verification, can aid in refining subtypes and identifying rare transitional forms. Such efforts will enable stratification of surgical risk, correlation with clinical outcomes, and potentially automated pattern recognition using machine learning on imaging datasets.

### 7.5 Author’s perspective on classification sufficiency

Based on comparative analysis of both fetal and adult populations, the present author asserts that the existing classification systems proposed in 2019 and 2021 are not only anatomically exhaustive, but also clinically applicable without modification. The developmental classification (2021) accounts for transitional morphologies, while the adult framework (2019) supports surgical planning and imaging interpretation with precision. Rather than introducing a new nomenclature, this chapter’s proposed unified model serves as a translational bridge, confirming that the original systems remain sufficient and validated by current literature and clinical evidence.

## 8 Future directions

Despite the progress made in understanding the anatomical variability and clinical implications of the FLT, several research avenues remain to be explored. A systematic approach combining anatomical, imaging, and clinical outcome data is essential for the translational advancement of FLT-related diagnostics and therapeutics.

First, prospective imaging studies using high-resolution MRI and dynamic US should aim to validate the unified classification system across diverse populations. These studies would benefit from histological and intraoperative correlation to distinguish minor fascial variants and fusion patterns that remain elusive on imaging alone.

Second, the development of standardized imaging protocols and reporting language specific to FLT variants would support reproducibility and interdisciplinary communication. Integration of classification types into radiology reports especially in cases of chronic lateral ankle pain, tendon graft planning, or unexplained foot deformities could improve diagnostic precision and surgical outcomes.

Third, clinical registries focusing on procedures involving the FLT such as tendon harvesting, tendoscopic decompression, or reconstructive transfers should be established. Subtype-stratified analyses could elucidate the impact of variant anatomy on postoperative outcomes, rehabilitation time, and risk of complications.

Fourth, a promising direction involves the application of artificial intelligence to FLT anatomy. Pattern recognition algorithms trained on large imaging datasets could eventually automate the detection of insertional variants or predict surgical difficulty based on preoperative scans.

Lastly, comparative anatomical and evolutionary studies should continue to explore the developmental origins and functional adaptations of the FLT. Integration with developmental biology and phylogenetics could yield insights into why certain variants persist or regress and how they relate to locomotor mechanics across species.

## 9 Conclusion

The fibularis longus tendon, though historically underappreciated in anatomical discourse, exhibits substantial morphological variation that bears direct clinical relevance. This review has established that the previously published fetal and adult classification systems by Olewnik (2019), Olewnik et al. (2021) remain comprehensive, valid, and clinically applicable. Their proposed unification provides a cohesive framework for interpreting imaging findings, planning surgical procedures, and anticipating anatomical complexity.

By bridging ontogenetic and clinical perspectives, this manuscript underscores the importance of structured classification in improving both diagnostic accuracy and surgical safety. The integration of anatomical knowledge into radiological and operative workflows holds promise for enhancing outcomes in procedures involving the lateral and plantar compartments of the foot.

In conclusion, continued investigation into the fibularis longus tendon will not only refine our anatomical understanding but also pave the way for precision medicine approaches in lower limb surgery. Through collaborative research and technological integration, the anatomy of the FLT may serve as a model for future advances in musculoskeletal diagnostics and therapeutics.
